# Comparative Evaluation and Measure of Accuracy of ELISAs, CLIAs, and ECLIAs for the Detection of HIV Infection among Blood Donors in China

**DOI:** 10.1155/2020/2164685

**Published:** 2020-08-14

**Authors:** Le Chang, Junpeng Zhao, Fei Guo, Huimin Ji, Lu Zhang, Xinyi Jiang, Lunan Wang

**Affiliations:** ^1^National Center for Clinical Laboratories, Beijing Hospital, National Center of Gerontology, Institute of Geriatric Medicine, Chinese Academy of Medical Sciences, Beijing, China; ^2^Beijing Engineering Research Center of Laboratory Medicine, Beijing Hospital, Beijing, China; ^3^Graduate School, Peking Union Medical College, Chinese Academy of Medical Sciences, Beijing, China; ^4^Shenzhen Blood Center, Shenzhen, China

## Abstract

**Background:**

Enzyme-linked immunosorbent assay (ELISA) is the only serological method approved for blood screening in China. Automated chemiluminescence immunoassay (CLIA) and electrochemiluminescence immunoassay (ECLIA) had been used in clinical laboratories but not applied to screen HIV among blood donors. This study aimed to evaluate the performance of ELISA, CLIA, and ECLIA, focusing on the feasibility of CLIA/ECLIA for blood screening.

**Method:**

1029 blood donations from 14 blood centers screened by ELISA were enrolled in the study. All plasma samples were tested by eight ELISA assays in 16 blood centers, followed by the detection of CLIA and ECLIA methods in the National Center for Clinical Laboratories (NCCL), further confirmed by nucleic acid testing (NAT) and Western blot (WB).

**Results:**

Of 1029 samples, 136 were confirmed as HIV positive. CLIA and ECLIA assay had similar sensitivities with ELISAs but showed higher specificity (CLIA: 99.1%, 885/893; ECLIA: 99.0%, 884/893), concordance rate (CLIA: 99.2%, 1021/1029; ECLIA: 99.1%, 1020/1029), and positive predictive value (PPV) (CLIA: 94.4%, 136/144; ECLIA: 93.8%, 136/145) than most of ELISA kits (>5 ELISAs) (*P* < 0.05). Kappa values of CLIA (0.967) and ECLIA (0.963) were the highest among all the serologic assays. Among 451 samples with initial ELISA reactivity, 315 were negatives, of which 307 (97.5%) and 306 (97.1%) were detected as nonreactive by CLIA (8 nonspecific reactions) and ECLIA (9 nonspecific reactions), respectively.

**Conclusion:**

Compared with ELISA, CLIA and ECLIA are more specific and accurate in detecting HIV antibody/antigen and can keep more nonspecifically reactive donors detected by ELISA. CLIA and ECLIA can be used for the improvement of serological blood screening strategy to avoid the unnecessary loss of blood donors.

## 1. Background

Ever since the first identification of HIV infectious case in Ruili City, Yunnan province, in 1985, HIV has spread numerically and geographically throughout China. According to the updated report, approximately 849,602 people are living with HIV and 262,442 individuals died of HIV-associated diseases in China [1]. The epidemic of HIV has been shifted from high risk population into the general population, including blood donors in China [2,3]. The prevalence of HIV among blood donors in China is about 10.33 in 100,000 between 2000 and 2009 [4]. Therefore, HIV screening is essential to ensure the strict safety of blood supply.

At present, NAT and ELISA are routinely applied to screen HIV infection. ELISA is the only approved serological assay for HIV screening among blood donations in China. The assay detects the presence of a ligand (commonly a protein) in a liquid sample through a solid-phase enzyme immunoassay (EIA) using antibodies directed against the protein to be measured [5]. The amounts of HIV antigens or antibodies are measured by the intensity of color produced by reaction between conjugate and substrate. CLIA and ECLIA methods use recombinant HIV antigens or antibodies coated paramagnetic microparticles that bind to HIV antigens or antibodies in plasma labelled recombinant HIV antigens or antibodies coated particles as conjugate. Antibody or antigen concentration is determined by the emitted light of antigen-antibody reaction and measured using light reader [6,7].

CLIA and ECLIA, including sample preprocessing system and result analysis system from the same manufacturers, are fully automated and self-contained platforms which minimize operator involvement and have good reproducibility and partly avoid the false positive/negative brought by operator factors [8–10]. However, even if using the same kit in automatic ELISA platform in different Chinese blood centers, the preprocessing system, incubation and washing system, and microplate reader could be completely different, due to the open system of ELISA devices. The assembled automatic ELISA system is hard to ensure the standardization of results between different blood centers. Hence, the suitability for ELISA is defined by different parameters and may be further affected by other factors which are based on open system. The diversity of the virus including the prevalence of groups and subtypes and circulating recombinant forms can influence the effectiveness of the diagnosis [11].

The current study aimed to compare the performance between eight ELISAs and CLIA/ECLIA and discuss whether CLIA/ECLIA can be used for blood screening in China.

## 2. Materials and Methods

### 2.1. HIV Confirmatory Algorithm and Study Design

**Figure 1:**
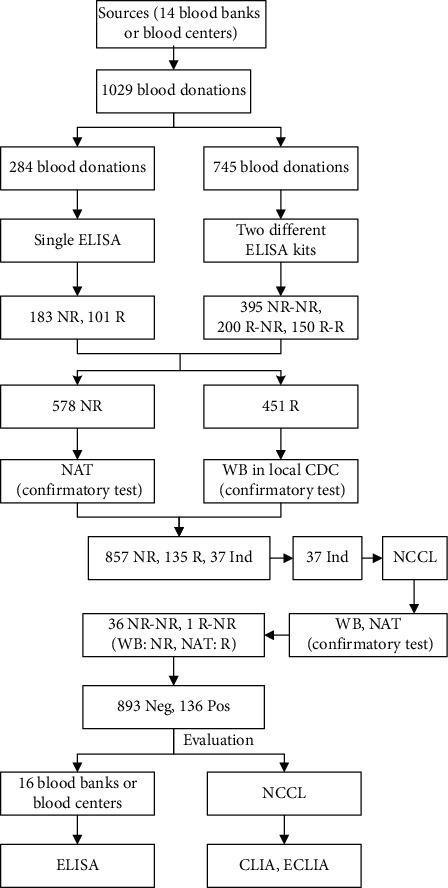
The study route. NR: nonreactive, R: reactive, Pos: positive, Neg: negative, WB: Western blot, CLIA: Abbott ARCHITECT HIV Ag/Ab Combo, ECLIA: Roche Elecsys HIV combi PT, and NAT: nucleic acid testing of Cobas TaqScreen MPX test v2.0.

From March 2015 to September 2015, 1029 blood donations detected as HIV reactive using one or two ELISAs which were collected from 14 blood centers or blood banks and were screened for HIV. The number of blood donations was equal to the number of blood donors. All blood donations underwent HIV confirmatory algorithm: all the initial negative samples were performed NAT in the National Center for Clinical Laboratories (NCCL), and initial positives were tested by Western blot (WB) by local centers for disease control and prevention (CDC); the samples with indeterminate results of WB were further confirmed using WB and NAT in NCCL. Then, all the samples were sent to 16 blood screening laboratories for ELISA evaluation using one or two of the eight assays with automation systems; furthermore, CLIA and ECLIA screening among all the blood donations were performed in NCCL (Table 1). The results of the same ELISA reagent used by different laboratories in the same sample were not shown. The final result of each ELISA assay among 1029 blood donations was determined by more than half the results of different blood centers using the same ELISA. The study route was shown in Figure 1.

### 2.2. Statistical Analyses

SPSS 21.0 software was used for statistical analysis. Chi-square tests were performed on all the examined outcomes to compare sensitivity, specificity, concordance rate, positive predictive value (PPV), and negative predictive value (NPV) between CLIA, ECLIA, and ELISAs; *P* < 0.05 was considered statistically significant. Sensitivity was the proportion of true positives among donations with serologic reactivity; specificity was calculated using the number of true negatives divided by nonreactive results in serologic assays. Concordance rate (accuracy) will be performed and calculated through the percentage of true positives and negatives among 1029 blood donations. PPV and NPV are the proportions of positive and negative results in diagnostic tests that are true positive and true negative results, respectively.

## 3. Results

Of 1029 donations, 136 were confirmed as HIV positive and 893 were HIV negative. PPV of initial ELISA test (30.2%) had a significant difference with CLIA (94.4%) and ECLIA (93.8%), respectively (*P* < 0.05) (Table 2). Among 451 samples with initial ELISA reactivity, 315 were negatives, of which 307 (97.5%) and 306 (97.1%) were detected as nonreactive by CLIA (8 nonspecific reactions) and ECLIA (9 nonspecific reactions), respectively.

As shown in Table 3, the sensitivity of CLIA or ECLIA had no significant difference with 8 ELISAs (*P* < 0.05), only two or three samples were missed by several ELISAs (not shown), but these samples were detected by CLIA, ECLIA, and NAT. The CLIA and ECLIA showed higher specificities and PPVs than most of ELISAs (Wantai, KHB, Bio-Rad, Wantai [4^th^], Murex [4^th^], Livzon [4^th^]), *P* < 0.05. Collectively, no significant differences found in sensitivity and NPV between 8 ELISAs and CLIA/ECLIA (*P* < 0.05); Kappa values of CLIA (0.967) and ECLIA (0.963) were the highest among all the serologic assays. However, ELISA evaluations in some laboratories (HN, JN, LN) showed lower sensitivities and NPVs than CLIA and ECLIA (*P* < 0.05). InTec and Livzon ELISAs had similar specificities/PPVs/concordance rates to CLIA and ECLIA (*P* < 0.05). However, among the five HIV blood screening laboratories using InTec, ELISA results in HLJ and LN showed lower specificities and concordance rates, in comparison to CLIA/ECLIA (*P* < 0.05). In addition, CLIA and ECLIA had higher PPV and concordance rate than InTec ELISAs tested by LN and CQ, respectively, *P* < 0.05. Besides, of 2 blood centers using Livzon, the ELISAs tested by HZ and ZH were less specific than CLIA/ECLIA, and ELISA screening in ZH had lower PPV than CLIA/ECLIA.

## 4. Discussion

ELISA is the only approved serological assay for HIV screening among blood donations in China. CLIA and ECLIA have been proved to be at least as specific and sensitive as common assays in serological detection of many infectious diseases [12–15] but not applied for HIV blood screening in China. CLIA and ECLIA have been used for routine screening among blood donors in the US [16], Australia [17], Sweden [18], and Italy [10] for many years. Hence, evaluation of ELISA, CLIA, and ECLIA among blood donations is critical for the development of HIV serological screening strategy. To date, this comparative study firstly investigated the performance of 8 ELISA kits (including all ELISA kits for HIV blood screening in China) for the detection of HIV and comparing CLIA, ECLIA, and 8 ELISAs among blood donors. The sources of study samples were comprehensive, including 14 blood screening laboratories. Additionally, less comparative evaluation measure of accuracy between CLIA, ECLIA, and ELISA among blood donors focused on the feasibility of CLIA/ECLIA for blood screening and the improvement of serological screening strategy in China.

Using one or two HIV ELISA kits followed with NAT were required by Chinese Blood Bank Technical Operation Procedure (2015 version) to screen HIV in blood banks. The majority of the samples that tested initial ELISA reactive for HIV were nonspecific reactivity in the present study. This phenomenon may be related to the low prevalence of HIV [19], apart from the factors of reagent and laboratories. The previous study reported that most of donations were discarded and donors were deferred for nonspecific reactivity [20]. According to an official investigation report about clinical blood transfusion, the growth rate of blood supply is still lower than the rate of growing clinical demand for transfusion [21]. ECLIA (97.1%, 306/315) and CLIA (97.5%, 307/315) methods could reduce 97% nonspecific reactions produced by ELISAs (Table 2). Hence, CLIA and ECLIA can be used for blood screening to avoid unnecessary loss of blood donations and also can be used for blood reentry [22].

In the study, we observed a high agreement in HIV antigen/antibody screening between CLIA and ECLIA, similar to Italy's study [10]. Previous studies indicated CLIA with high sensitivity, specificity, and high degree of automation for HIV antibody-antigen testing can be used for the detection of HIV infection [15,23,24]. 4^th^ generation serological assays can detect HIV antigen and antibody, while 3^rd^ generation serological assays only detect HIV antibody. The results showed that the specificity, concordance rate, and PPV of 3^rd^ generation ELISA kits were higher than 4^th^ generation ELISA kits, possibly due to higher sensitivity and nonspecific reactivity of 4^th^ generation ELISA kits [25,26]. However, CLIA and ECLIA, as 4^th^ generation serological assays, are more specific and accurate to detect HIV antigen/antibody among blood donations, compared with the most of ELISAs (3^rd^ and 4^th^ generation ELISA). Note that specificity of CLIA and ECLIA was significantly higher than most of the ELISA kits, which demonstrated that ELISA may lead to more loss of donations due to the nonspecific reactions.

Among eight ELISAs, only InTec and Livzon were not different from CLIA/ECLIA about specificity/PPV/concordance rate. Nevertheless, InTec ELISAs tested by HLJ, LN, and CQ and Livzon ELISAs tested by HZ and ZH had lower specificities, PPVs, and/or concordance rates than ECLIA and CLIA, suggesting that using the same ELISA reagent to test the same sample in different laboratories may produce different results. It indicated that the open system assay (ELISA) may lead to less than optimum results and the performance of the assay being evaluated is considered to be lower than it actually is, due to the requirement of high level of operator involvement and the overall performance of the assay itself [27,28]. The assembly automatic ELISA system may lead to bias of result in the same ELISA kit and the same sample between different laboratories. ELISA kits used in the blood centers can present a great variability depending on the supplier. For CLIA and ECLIA methods, there are few opportunities for error; the systems and assays can only be run as intended by the manufacturer, controlled by the system software.

In summary, significant differences in sensitivity, specificity, concordance rate, PPV, and NPV between some ELISAs and CLIA/ECLIA were found in several blood centers.

At present, all blood centers must use ELISA as serological assay for HIV blood screening; the performance of ELISA about sensitivity was decent for blood supply. Only two or three positive samples were missed by ELISAs, while none was missed by NAT. Consequently, HIV screening strategy in China can ensure the blood safety. The sensitivity of ELISA met the detection of HIV among blood donors, which is the primary factor of blood screening assay, but specificity and feasibility must also be considered, the balance point between sensitivity, specificity, and feasibility being determined by each transfusion service. CLIA and ECLIA had been used in clinical laboratories in China, with higher specificity and lower operator involvement than ELISA, but the higher cost of CLIA and ECLIA limited their application in blood centers. However, the benefit of them for the health system must be taken into account, since their sensitivity and efficiency have been scientifically proven. After large-scale industrial production and policy support, the cost of CLIA/ECLIA will be under control. Furthermore, the parts of automatic ELISA system were different from ELISA manufacturers and blood centers, which were hard to standardize. In the future, CLIA or ECLIA may be approved by local authorities and used as a second confirmation step or routine screening for the detection of HIV infections among Chinese blood donors. If not all laboratories can use these techniques, centralized labs could perform the tests.

## 5. Conclusions

In summary, compared with ELISA, CLIA and ECLIA of full automation and closed system are more specific and accurate to detect HIV antibody/antigen among blood donations, which can keep a large number of blood donations with nonspecific ELISA reactions. The feasibility of CLIA and ECLIA is decent for blood screening, which may be approved as serological screening tool among blood donations to avoid the unnecessary loss of blood donors in China.

The limitation of the research is that CLIA/ECLIA and ELISA cannot simultaneously be performed by blood centers, due to no CLIA and ECLIA platform in blood centers. Comprehensive and comparative study of HIV serological screening about ELISA, CLIA, and ECLIA (CLIA and ECLIA: Including Abbott, Roche, and domestic manufacturers) among blood donors in China is already in progress.

## Figures and Tables

**Table 1:** Blood screening laboratories of study participation and their commercially available HIV serology kits.

Code	Blood screening labs	HIV reactive (*n* = 451)	HIV nonreactive (*n* = 578)	HIV serology kits	
1	SZ	73	50	Bio-Rad HIV Ab-Ag (4^th^)	—
2	XY	14	9	InTec HIV Ab	Wantai HIV Ab
3	HB	26	18	Murex HIV Ab-Ag (4^th^)	Wantai HIV Ab-Ag (4^th^)
4	JS	32	50	InTec HIV Ab	Wantai HIV Ab-Ag (4^th^)
5	LN	19	50	KHB HIV Ab	InTec HIV Ab
6	HLJ	29	76	Bio-Rad HIV Ab-Ag (4^th^)	InTec HIV Ab
7	SD	44	50	InTec HIV Ab	Murex HIV Ab-Ag (4^th^)
8	TJ	70	49	Murex HIV Ab-Ag (4^th^)	Wantai HIV Ab-Ag (4^th^)
9	HN	25	112	Wantai HIV Ab	-
10	CQ	31	18	Bio-Rad HIV Ab-Ag (4^th^)	InTec HIV Ab
11	YN	8	15	Bio-Rad HIV Ab-Ag (4^th^)	KHB HIV Ab
12	JL	48	11	KHB HIV Ab	Wantai HIV Ab-Ag (4^th^)
13	HZ	3	21	Livzon HIV Ab	—
14	TZ	29	49	Bio-Rad HIV Ab-Ag (4^th^)	Murex HIV Ab-Ag (4^th^)
15	BJ	—	—	Bio-Rad HIV Ab-Ag (4^th^)	Wantai HIV Ab
16	ZH	—	—	Livzon HIV Ab-Ag (4^th^)	—
17	JN	—	—	KHB HIV Ab	Wantai HIV Ab-Ag (4^th^)
18	CZ	—	—	Murex HIV Ab-Ag (4^th^)	—
19	NCCL	—	—	Abbott ARCHITECT HIV Ag/Ab Combo^§^ (4^th^)	Roche Elecsys HIV combi PT^†^ (4^th^)

a: ^§^ CLIA,^†^ ECLIA, and other HIV serology kits are ELISAs. b: 4^th^: fourth-generation ELISA kits. Other HIV serology kits are third-generation ELISA kits. ELISAs: Wantai Diagnostic Kit for antibody to human immunodeficiency virus (Beijing Wantai Biological Pharmacy, Beijing, China) (Wantai), Wantai Diagnostic Kit for antibody and antigen to human immunodeficiency virus (Beijing Wantai Biological Pharmacy, Beijing, China) (Wantai (4^th^)), Beijing, China; KHB Diagnostic Kit for antibody to human immunodeficiency virus (Shanghai Kehua Bioengineering Co., Shanghai, China) (KHB), InTec Diagnostic Kit for antibody to human immunodeficiency virus (InTec Products, Inc., Xiamen, China) (InTec), Livzon Diagnostic Kit for antibody to human immunodeficiency virus (Zhuhai Livzon Diagnostics Inc., Zhuhai, China) (Livzon), Livzon Diagnostic Kit for antibody and antigen to human immunodeficiency virus (Zhuhai Livzon Diagnostics Inc., Zhuhai, China) (Livzon (4^th^)), Murex HIV Ag/Ab combination (Diasorin, Saluggia, Italy) (Murex (4^th^)), Bio-Rad GS HIV Combo Ag/Ab EIA (Bio-Rad, Marnes la Coquette, France) (Bio-Rad (4^th^)). c: blood centers (codes 1–14) were sources of study samples. d: blood centers (codes 3–18) performed HIV screening for all the samples using one or two ELISA kits. CLIA and ECLIA were conducted by NCCL.

**Table 2:** The NPV and PPV of initial ELISA results for HIV blood screening.

HIV screening	Single ELISA kit (284)		Two different ELISA kits (745)			ELISA (1029)			ECLIA		CLIA	
Status	Nonreactive	Reactive	Dual nonreactive	Single reactive	Dual reactive	Nonreactive	Reactive	Sum	Nonreactive	Reactive	Nonreactive	Reactive
	183	101	395	200	150	578	451	1029	884	145	885	144
True negatives	183	71	395	198	46	578	315	893	884	9	885	8
True positives	0	30	0	2	104	0	136	136	0	136	0	136
NPV^§^	100% (183/183)	—	100% (395/395)	—	—	100% (578/578)	—	100% (578/578)	100% (884/884)	—	100% (885/885)	—
PPV^§^	—	29.7% (30/101)	—	1.0% (2/200)	69.3% (104/150)	—	30.2% (136/451)	30.2% (136/451)	—	93.8% (136/145)	—	94.4% (136/144)

^§^: positive predictive value (PPV) and negative predictive value (NPV) are the proportions of positive and negative results in statistics and diagnostic tests that are true positive and true negative results, respectively.

**Table 3:** The performance of serology kits for HIV screening^§^.

Blood screening laboratories	HIV serology kits	Sensitivity *n* = 136 (%), 95% CI	Specificity *n* = 893 (%), 95% CI	Concordance rate^||^*n* = 1029 (%), 95% CI	PPV, 95% CI	NPV, 95% CI	Kappa (*κ*)
BJ	Wantai	134 (98.5%), 96.5%–100.5%	859 (96.2%)^*∗∗*^^††^, 95.0%–97.4%	993 (96.5%)^*∗∗*^^††^, 95.4%–97.6%	79.8%^*∗∗*^^††^, 73.7%–85.8%	99.8%, 99.4%–100.1%	0.861
HN	Wantai	131 (96.3%)^*∗*^^†^, 93.1%–99.5%	839 (94.0%)^*∗∗*^^††^, 92.5%–95.5%	970 (94.3%)^*∗∗*^^††^, 92.9%–95.7%	70.8%^*∗∗*^^††^, 64.3%–77.3%	99.4%^*∗*^^†^, 98.9%–99.9%	0.783
	Wantai^§^	134 (98.5%), 96.5%–100.5%	861(96.4%)^*∗∗*^^††^, 95.2%–97.6%	995 (96.7%)^*∗∗*^^††^, 95.6%–97.8%	80.7%^*∗∗*^^††^, 74.8%–86.7%	99.8%, 99.4%–100.1%	0.868
YN	KHB	134 (98.5%), 96.5%–100.5%	872 (97.6%)^*∗*^^†^, 96.6%–98.6%	1006 (97.8%)^*∗*^^††^, 96.9%–98.7%	86.5%^*∗*^^†^, 81.1%–91.8%	99.8%, 99.5%–100.1%	0.908
JN	KHB	131 (96.3%)^*∗*^^†^, 93.1%–99.5%	868 (97.2%)^*∗∗*^^††^, 96.1%–98.3%	999 (97.1%)^*∗∗*^^††^, 96.1%–98.1%	84.0%^*∗∗*^^††^, 78.2%–89.7%	99.4%^*∗*^^†^, 98.9%–99.9%	0.880
LN	KHB	129 (94.9%)^*∗∗*^^††^, 91.2%–98.6%	843 (94.4%)^*∗∗*^^††^, 92.9%–95.9%	972 (94.5%)^*∗∗*^^††^, 95.4%–97.6%	72.1%^*∗∗*^^††^, 65.5%–78.6%	99.2%^*∗∗*^^††^, 98.6%–99.8%	0.787
	KHB^§^	134 (98.5%), 96.5%–100.5%	873 (97.8%)^*∗*^^†^, 96.8%–98.8%	1007 (97.9%)^*∗*^^†^, 97.0%–98.8%	87.0%^*∗*^^†^, 81.7%–92.3%	99.8%, 99.5%–100.1%	0.912
SD	InTec	133 (97.8%), 95.3%–100.3%	879 (98.4%), 97.6%–99.2%	1012 (98.3%), 97.5%–99.1%	90.5%, 85.8%–95.2%	99.7%, 99.3%–100.0%	0.930
JS	InTec	133 (97.8%), 95.3%–100.3%	880 (98.5%), 97.7%–99.3%	1013 (98.4%), 97.6%–99.2%	91.1%, 86.5%–95.7%	99.7%, 99.3%–100.0%	0.934
HLJ	InTec	133 (97.8%), 95.3%–100.3%	875 (98.0%)^†^, 97.1%–98.9%	1008 (98.0%)^*∗*^^†^, 97.1%–98.9%	88.1%, 82.9%–93.2%	99.7%, 99.3%–100.0%	0.915
LN	InTec	132 (97.1%)^*∗*^^†^, 94.3%–99.9%	872 (97.6%)^*∗*^^†^, 96.6%–98.6%	1004 (97.6%)^*∗∗*^^††^, 96.7%–98.5%	86.3%^*∗*^^†^, 80.8%–91.7%	99.5%, 99.1%–100.0%	0.899
CQ	InTec	133 (97.8%), 95.3%–100.3%	876 (98.1%), 97.2%–99.0%	1009 (98.1%)^*∗*^^†^, 97.3%–98.9%	88.7%, 83.6%–93.7%	99.7%, 99.3%–100.0%	0.919
	InTec^§^	133 (97.8%), 95.3%–100.3%	881 (98.7%), 98.0%–99.4%	1014 (98.5%), 97.8%–99.2%	91.7%, 87.3%–96.2%	99.7%, 99.3%–100.0%	0.938
HZ	Livzon	133 (97.8%), 95.3%–100.3%	875 (98.0%)^†^, 97.1%–98.9%	1008 (98.0%)^*∗*^^†^, 97.1%–98.9%	88.1%, 82.9%–93.2%	99.7%, 99.3%–100.0%	0.915
ZH	Livzon	133 (97.8%), 95.3%–100.3%	873 (97.8%)^*∗*^^†^, 96.8%–98.8%	1006 (97.8%)^*∗*^^††^, 96.9%–98.7%	86.9%^*∗*^^†^, 81.6%–92.2%	99.7%, 99.3%–100.0%	0.907
	Livzon^§^	133 (97.8%), 95.3%–100.3%	876 (98.1%), 97.2%–99.0%	1009 (98.1%)^*∗*^^†^, 97.3%–98.9%	88.7%, 83.6%–93.7%	99.7%, 99.3%–100.0%	0.919
SD	Bio-Rad (4^th^)	136 (100%), 100.0%–100.0%	795 (89.0%)^*∗∗*^^††^, 87.0%–91.0%	931 (90.5%)^*∗∗*^^††^, 88.7%–92.3%	58.1%^*∗∗*^^††^, 51.8%–64.4%	100%, 100.0%–100.0%	0.682
SZ	Bio-Rad (4^th^)	136 (100%), 100.0%–100.0%	795 (89.0%)^*∗∗*^^††^, 87.0%–91.0%	931 (90.5%)^*∗∗*^^††^, 88.7%–92.3%	58.1%^*∗∗*^^††^, 51.8%–64.4%	100%, 100.0%–100.0%	0.682
BJ	Bio-Rad (4^th^)	136 (100%), 100.0%–100.0%	798 (89.4%)^*∗∗*^^††^, 87.4%–91.4%	934 (90.8%)^*∗∗*^^††^, 89.0%–92.6%	58.9%^*∗∗*^^††^, 52.6%–65.2%	100%, 100.0%–100.0%	0.689
HLJ	Bio-Rad (4^th^)	136 (100%), 100.0%–100.0%	797 (89.2%)^*∗∗*^^††^, 87.2%–91.2%	933 (90.7%)^*∗∗*^^††^, 88.9%–92.5%	58.6%^*∗∗*^^††^, 52.3%–64.9%	100%, 100.0%–100.0%	0.687
	Bio-Rad (4^th^)^§^	136 (100%), 100.0%–100.0%	812 (90.9%)^*∗∗*^^††^, 89.0%–92.8%	948 (92.1%)^*∗∗*^^††^, 90.5%–93.7%	62.7%^*∗∗*^^††^, 56.3%–69.1%	100%, 100.0%–100.0%	0.726
JL	Wantai (4^th^)	135 (99.3%), 97.9.0%–100.7%	843 (94.4%)^*∗∗*^^††^, 92.9%–95.9%	978 (95.0%)^*∗∗*^^††^, 93.7%–96.3%	73.0%^*∗∗*^^††^, 66.6%–79.3%	99.9%, 99.7%–100.1%	0.813
JS	Wantai (4^th^)	135 (99.3%), 97.9.0%–100.7%	863 (96.6%)^*∗∗*^^††^, 95.4%–97.8%	998 (97.0%)^*∗∗*^^††^, 96.0%–98.0%	81.8%^*∗∗*^^††^, 76.0%–87.7%	99.9%, 99.7%–100.1%	0.88
TJ	Wantai (4^th^)	133 (97.8%), 95.3%–100.3%	853 (95.5%)^*∗∗*^^††^, 94.1%–96.9%	986 (95.8%)^*∗∗*^^††^, 94.6%–97.0%	76.9%^*∗∗*^^††^, 70.6%–83.1%	99.6%, 99.3%–100.0%	0.837
HB	Wantai (4^th^)	134 (98.5%), 96.5%–100.5%	854 (95.6%)^*∗∗*^^††^, 94.3%–96.9%	988 (96.0%)^*∗∗*^^††^, 94.8%–97.2%	77.5%^*∗∗*^^††^, 71.3%–83.7%	99.8%, 99.4%–100.1%	0.844
	Wantai (4^th^)^§^	135 (99.3%), 97.9.0%–100.7%	863 (96.6%)^*∗∗*^^††^, 95.4%–97.8%	998 (97.0%)^*∗∗*^^††^, 96.0%–98.0%	81.8%^*∗∗*^^††^, 76.0%–87.7%	99.9%, 99.7%–100.1%	0.880
TZ	Murex (4^th^)	135 (99.3%), 97.9.0%–100.7%	833 (93.3%)^*∗∗*^^††^, 91.7%–94.9%	968 (94.1%)^*∗∗*^^††^, 92.7%–95.5%	69.2%^*∗∗*^^††^, 62.8%–75.7%	99.9%, 99.6%–100.1%	0.782
CZ	Murex (4^th^)	135 (99.3%), 97.9.0%–100.7%	834 (93.4%)^*∗∗*^^††^, 91.8%–95.0%	969 (94.2%)^*∗∗*^^††^, 92.8%–95.6%	69.6%^*∗∗*^^††^, 63.1%–76.0%	99.9%, 99.6%–100.1%	0.785
HB	Murex (4^th^)	135 (99.3%), 97.9.0%–100.7%	849 (95.1%)^*∗∗*^^††^, 93.7%–96.5%	984 (95.6%)^*∗∗*^^††^, 94.4%–96.8%	75.4%^*∗∗*^^††^, 69.1%–81.7%	99.9%, 99.7%–100.1%	0.832
	Murex (4^th^)^§^	135 (99.3%), 97.9.0%–100.7%	842 (94.3%)^*∗∗*^^††^, 92.8%–95.8%	977 (94.9%)^*∗∗*^^††^, 93.6%–96.2%	72.6%^*∗∗*^^††^, 66.2%–79.0%	99.9%, 99.7%–100.1%	0.809
ZH	Livzon (4^th^)	135 (99.3%), 97.9.0%–100.7%	869 (97.3%)^*∗∗*^^††^, 96.2%–98.4%	1004 (97.6%)^*∗∗*^^††^, 96.7%–98.5%	84.90%^*∗*^^††^, 79.4%–90.4%	99.90%, 99.7%–100.1%	0.901
NCCL	ECLIA	136 (100%), 100.0%–100.0%	884 (99.0%), 98.4%–99.6%	1020 (99.1%), 98.5%–99.7%	93.8%, 89.9%–97.7%	100%, 100.0%–100.0%	0.963
NCCL	CLIA	136 (100%), 100.0%–100.0%	885 (99.1%), 98.5%–99.7%	1021 (99.2%), 98.7%–99.7%	94.4%, 90.7%–98.2%	100%, 100.0%–100.0%	0.967

^§^The final result of each ELISA among 1029 blood donations was determined by more than half results of different blood centers using the same ELISA and S/CO if the number of reactive and nonreactive results was equal. ^||^The proportion of true results among samples screened by HIV serology kits. ^*∗*^*P* < 0.05, ^*∗∗*^*P* < 0.01, ^†^*P* < 0.05, ^††^*P* < 0.01, ELISA, and ECLIA: the values (^∗^) were compared with ECLIA (Roche Elecsys HIV combi PT); ELISA and CLIA: the values (^†^) were compared with CLIA (Abbott ARCHITECT HIV Ag/Ab Combo).

## Data Availability

The data used to support this study are available from the corresponding author upon request.

## Abbreviations

HIV: Human immunodeficiency virus
ELISA: Enzyme-linked immunosorbent assay
CLIA: Chemiluminescence immunoassay
ECLIA: Electrochemiluminescence immunoassay
NCCL: National Center for Clinical Laboratories
NAT: Nucleic acid testing
WB: Western blot
AIDS/STD: Acquired immunodeficiency syndrome/sexually transmitted disease
EIA: Enzyme immunoassay
HBV: Hepatitis B virus
HCV: Hepatitis C virus
ALT: Alanine transaminase
S/CO: Signal to cut-off values
PPV: Positive predictive value
NPV: Negative predictive value
CDC: Center for Disease Control and Prevention
NAT: Nucleic acid testing.
